# Telomeres and Telomere Length: A General Overview

**DOI:** 10.3390/cancers12030558

**Published:** 2020-02-28

**Authors:** Nalini Srinivas, Sivaramakrishna Rachakonda, Rajiv Kumar

**Affiliations:** Division of Functional Genome Analysis, German Cancer Research Center, Im Neunheimer Feld 580, 69120 Heidelberg, Germany; n.srinivas@dkfz.de (N.S.); rpsrk@yahoo.com (S.R.)

**Keywords:** telomeres, shelterin complex, end replication, telomere maintenance mechanisms, *TERT* promoter mutations, telomere length heritability, genetic variants, cancer-risk

## Abstract

Telomeres are highly conserved tandem nucleotide repeats that include proximal double-stranded and distal single-stranded regions that in complex with shelterin proteins afford protection at chromosomal ends to maintain genomic integrity. Due to the inherent limitations of DNA replication and telomerase suppression in most somatic cells, telomeres undergo age-dependent incremental attrition. Short or dysfunctional telomeres are recognized as DNA double-stranded breaks, triggering cells to undergo replicative senescence. Telomere shortening, therefore, acts as a counting mechanism that drives replicative senescence by limiting the mitotic potential of cells. Telomere length, a complex hereditary trait, is associated with aging and age-related diseases. Epidemiological data, in general, support an association with varying magnitudes between constitutive telomere length and several disorders, including cancers. Telomere attrition is also influenced by oxidative damage and replicative stress caused by genetic, epigenetic, and environmental factors. Several single nucleotide polymorphisms at different loci, identified through genome-wide association studies, influence inter-individual variation in telomere length. In addition to genetic factors, environmental factors also influence telomere length during growth and development. Telomeres hold potential as biomarkers that reflect the genetic predisposition together with the impact of environmental conditions and as targets for anti-cancer therapies.

## 1. Introduction

**Figure 1:**
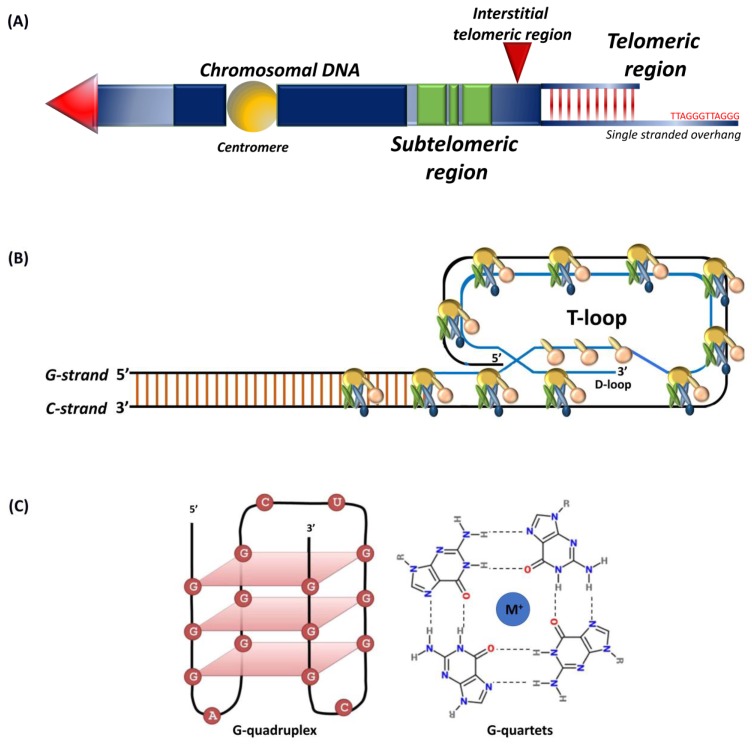
Schematic representation of (**A**) telomeres and subtelomeric regions, tandem nucleotide repeats at chromosomal ends that include a double-stranded region and a 50–300 nucleotide single-stranded guanine rich G-tail. Subtelomers (green) represent regions of genes interspersed within repeat elements and interstitial telomeric sequence (red arrow); (**B**) shelterin complex, the G-tail folds back into the duplex DNA to form the t-loop; (**C**) G-quadruplex structure, intramolecular G-quadruplex (left) built from G-quartets that are formed through cyclic Hoogsten hydrogen-bonding arrangement of four guanines with each other with G-tetrad structure on the right. Adapted from [23,24].

Telomeres are conserved tandem repeats at chromosomal ends that differ in length in diverse species [1,2,3,4,5]. Initially discovered in the extrachromosomal ribosomal DNA of *Tetrahymena thermophile*, the protozoan telomeres contain 20–70 hexameric TTGGGG tandem repeats [6]. The telomeres in yeast comprise of GGTTACA repeat sequences that extend up to 300 bp [7,8]. In plants, TTTAGGG repeats typically range between 2 to 100 kb, and certain protozoan and fungi carry short telomeres ranging between 18 to 600 bp [9,10]. In vertebrates, chromosomal ends consist of TTAGGG repeats with the longest telomeres being in rats and some strains of *Mus musculus* that extend up to 150 kb [5,7]. Human telomeres typically range between 10 to 15 kb [7,11,12]. Telomeres include proximal double-stranded and distal single-stranded regions (Figure 1A) with subtelomeres and interstitial sections separating repeats from the rest of the chromosome [13,14]. Telomeres, intrinsically unstable fragile sites, are stabilized through binding with so-called shelterin complex proteins [12,15,16].

Single-stranded 50–300 nucleotide guanine rich telomeric G-tail folds back into the duplex DNA to form a t-loop (Figure 1B) that resembles a large “lariat-like” structure [1,17,18]. The G-tail can also fold into a four-stranded helical structure known as the G-quadruplex (Figure 1C) that involves stacking of G-quartets and intra-molecular folding by overcoming kinetic barriers, with each quartet formed by the association of four guanines into a cyclic Hoogsten hydrogen-bonding arrangement [19,20]. Those compact and stable structures, besides forming a telomeric cap, inhibit access to telomerase [21]. Although the G-quadruplex structure in vivo has been observed by nuclear magnetic resonance, its biological function remains unknown [20,22].

## 2. Telomere-Associated Proteins

Telomeres are, in general, associated with three types of proteins that include nucleosomes, shelterin complex, and chromosomal transcription factors [13,25,26].

### 2.1. Nucleosomes

**Figure 2:**
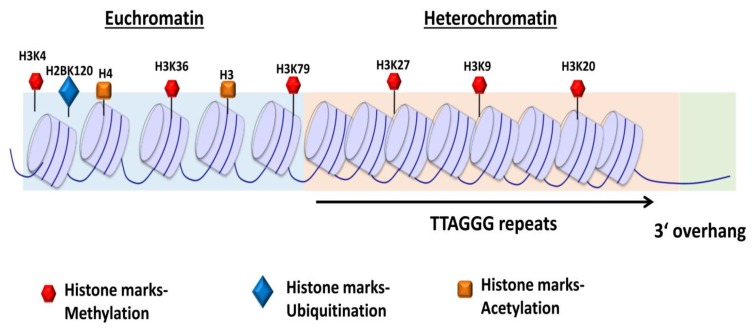
Schematic representation of chromatin structure and distribution of histone marks on telomeres. The telomeres are tightly packed into nucleosomes, the structural and functional units of chromatin. The euchromatin-associated and heterochromatin-associated histone marks are indicated. The euchromatin-associated marks include H4ac, H4K20me1, H3ac, H3K4me1/2/3, H3K36me2/3, H3K27ac, H3K79me3, and H2BK120ub. The heterochromatin-associated marks include H4K20me3, H3K9me3, and H3K27me3. Adapted from [34].

The telomeres, organized within tightly packed histone octamer composed nucleosomes (Figure 2), are stabilized through specific protein–protein and protein–DNA interactions between shelterin subunits and tandem repeat sequences [25,27]. Telomeres in higher eukaryotes are mainly heterochromatins enriched with histone 3 trimethylated at lysine 9 (H3K9me3) and histone 4 trimethylated at lysine 20 (H4K20me3) and heterochromatin protein (HP) isoforms [28,29,30]. The histone methyltransferases, SUV39H1 and SUV39H2, promote the methylation of H3K9 residues [31]. H3K9me3 recruits HP1 proteins, which are important for chromatin compaction through a high binding affinity site [29,32]. The heterochromatic region maintains telomeric structural integrity [29]. The loss of heterochromatic marks results in an open chromatin conformation, defective telomere function, aberrantly increased telomere length, and chromosomal instability [33].

Besides the routine post-translational modifications, histone proteins function in telomere capping, telomere transcription, homologous recombination at telomeres, cellular differentiation, and nuclear reprogramming [29,34]. The heterochromatin structure transcriptionally silences nearby genes, a phenomenon attributed to the telomere position effect (TPE) [34]. TPE mainly involves the shelterin protein, repressor and activator protein 1 (RAP1), and histone acetylase, SIRT6, a homolog of the yeast protein silent information regulator 2 (Sir2). RAP1 recruits SIRT6 protein, which on telomeres interact and promote hypo-acetylation of histone marks for active transcriptional repression of nearby genes [35].

### 2.2. Shelterin Complex

**Figure 3:**
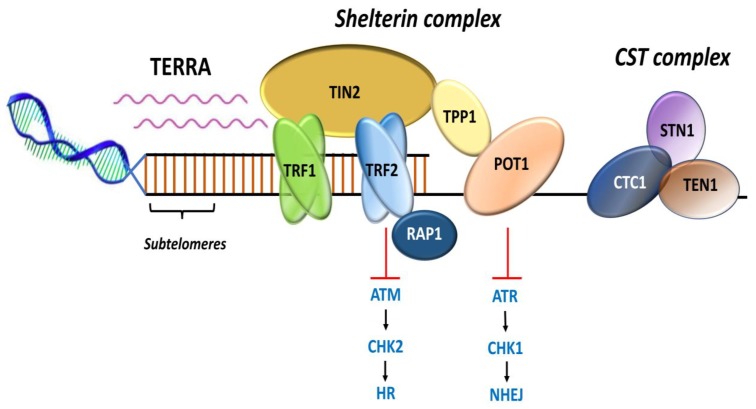
Representation of shelterin complex, heterotrimeric complex CST, and telomeric repeat containing RNA (TERRA). Shelterin complex comprises of six distinct protein subunits: telomeric-repeat-binding factor 1 and 2 (TRF1 and TRF2), TRF1-interacting nuclear protein 2 (TIN2), protection of telomeres 1 (POT1), POT1 and TIN2-interacting protein (TPP1), and repressor and activator protein 1 (RAP1). TRF1 and TRF2 bind the double-stranded DNA; POT1 binds the single-stranded 3′ G-overhang. TIN2 bridges TRF1 and TRF2 by binding to both the proteins simultaneously through independent domains and recruits TPP1–POT1 complex. RAP1 interacts with TRF2 to localize at the telomeres. CST complex is a heterotrimeric protein consisting of conserved telomere protection component 1 (CTC1), suppressor of cdc13 a (STN1), and telomeric pathway with STN1 (TEN1), which specifically localize to the single-stranded 3′ overhang and protect the telomeres by mediating DNA replication and telomerase regulation, independent of shelterin complex. Telomeric repeat containing RNA (TERRA) transcription initiates within subtelomeres in the direction of telomeres. TERRA is involved in regulating telomere capping and the maintenance of telomeres. Adapted from [13].

Shelterin complex comprises of six protein subunits [13]. Telomeric-repeat-binding factor 1 and 2 (TRF1 and TRF2) and protection of telomeres 1 (POT1) bind to DNA, and TRF1-interacting nuclear protein 2 (TIN2), TIN2-interacting protein (TPP1), and RAP1 act as adaptors (Figure 3) and mediate interactions among the constituents [5,36]. The shelterin complex functions as a dynamic unit in regulating telomere length, protects the chromosomal ends from being recognized as DNA damage, and represses DNA damage response (DDR) signals [13,37,38].

TRF1 and TRF2 that exist as homodimers bind to the double-stranded DNA, and POT1 binds to the single-stranded 3′ G-overhang [39,40]. TRF1 and TRF2 contain a TRF homology (TRFH) domain that mediates homodimerization and a Myb-type domain that specifically binds to the telomere duplex [40,41,42]. TRF1 and TRF2 both negatively regulate telomere length and promote efficient telomere replication [42]. The TRFH domain of TRF2 regulates the formation of the t-loop, whose assembly and disassembly is coordinated during the cell cycle by a phospho-switch [18,43,44]. TRF1 and TRF2 also suppress non-homologous end joining (NHEJ) and ataxia telangiectasia mutated (ATM)-dependent DNA damage signaling [39]. TIN2 bridges TRF1 and TRF2 by binding to both the proteins simultaneously through independent domains. The TRFH domain of TRF1 mediates the TIN2–TRF1 interaction and the TIN2–TRF2 interaction is mediated by a short motif in the hinge domain of TRF2 [42,45]. TIN2 further recruits TPP1 forming a triple complex—TIN2-TPP1-TRF2 [46]. This interaction provides a structural basis for shelterin bridge assembly [47].

POT1, that binds to single-stranded DNA with high specificity, contains two N-terminal oligonucleotide/oligosaccharide binding (OB) folds [13]. The first OB fold binds to the hexamer repeat at the beginning of the strand while the second OB fold binds and protects the 3′ G-overhang [48,49]. POT1 represses the ATM- and RAD3- related protein (ATR)-dependent signaling pathway and protects the telomere ends from fusion [50]. TPP1 binding remains essential for recruiting POT1 to the telomeres as those form heterodimers, which enhances the function of POT1 at the single-stranded 3′ end of telomeres [51,52]. While POT1 directly binds to single-stranded DNA, it indirectly interacts with the double-stranded DNA through association with TPP1 [46]. A biological role for TIN2 dependent on TPP1-POT1 has been suggested where its binding stabilizes the complex and promotes telomere processivity [53]. Accordingly, TIN2, along with TPP1-POT1, forms as a specialized telomeric single-stranded DNA binding sub-complex within the shelterin complex [41,53].

RAP1 does not bind directly to the DNA, but rather forms a complex with TRF2 and its Myb domain binds to the primary domain of TRF2 for suppressing telomeric homologous recombination [54,55]. The RAP1-TRF2 complex represses the localization of proteins such as the poly (ADP-ribose) polymerase 1 and SLX4 (SLX4 structure-specific endonuclease subunit) to the telomeres [55].

### 2.3. Other Telomere-Interacting Complexes

Several protein complexes, apart from the shelterin, contribute to telomere regulation and maintenance [56,57]. Those are either directly recruited to the telomeres or through interactions with the shelterin components [56]. CST, a heterotrimeric protein complex (Figure 3) consisting of conserved telomere protection component 1 (CTC1), suppressor of cdc13a (STN1), and telomeric pathway with STN1 (TEN1), localizes at single strand and functions in telomere capping and length regulation [58,59,60]. The CST complex interacts with DNA Polα-primase during telomere replication [58]. In vitro biochemical analysis has shown that CST unfolds G-quadruplex structures to facilitate replication through telomeres [61,62]. The complex has also been shown to localize with Polα at DNA damage sites and fill in double-stranded breaks through interaction with the shieldin complex, a 53BP1 effector complex involved in DDR [63]. The STN1-TEN1 subunit of CST complex functions in resolving replication fork during replication stress and regulates telomerase-mediated extension of the 3′ G-overhang [64,65].

Some of the proteins associated with telomeres are also involved in the DDR mechanism [57]. RecQ-family DNA helicases, Werner (WRN) and bloom (BLM), are recruited to the telomeres through TRF1 and TRF2 [66,67]. RecQ helicase proteins are involved in unwinding of G-quadruplex structure and initiation of DNA replication [68]. In addition, excision repair cross complementing associated with xeroderma pigmentosum group F (ERCC/XPF) mediates the 3′ overhang process; the recombination protein RAD51 and the helicase regulator of telomere length 1 (RTEL1) are involved in the replication and recombination of telomeric DNA [69,70].

### 2.4. Subtelomeres

Subtelomeres are transcriptionally active chromatin regions (Figure 1A) between main chromosomal sequences and telomeres [71]. The subtelomeric region constitutes two major zones: polymorphic patchworks of inter-chromosomal segmental duplication region and a chromosome specific non-duplicated region [72,73]. Segmental duplicated regions constitute about 5% of the human genome and cover 5 to 300 kb of terminal chromosome sequences [72]. Subtelomeres are packed into constitutive heterochromatin that mainly contains H3K9me3 heterochromatin marks and also harbors transcriptional start sites for telomeric repeat-containing RNAs (TERRA) [74,75,76]. TERRA transcription initiates from within subtelomeres (Figure 3) towards telomeres [76,77]. TERRAs, associated with heterochromatin marks such as HP1 and H3K9me3, actively participate in telomere maintenance/end protection and heterochromatin formation [78,79,80]. Transcription factors such as SNAIL1, involved in the epithelial-to-mesenchymal transition, control telomere transcription, and integrity by negatively regulating TERRA [81]. The segmental duplicated region of subtelomeres contains protein coding genes that vary in copy number and is located on different chromosomes, such as *WASH* at 9p, 2p, Xq/Yq, 1p, 15q, and 16p; immunoglobulin heavy chain genes at 14q; and olfactory receptor genes at 1p, 6p, 8p, 11p, 15q, 19p, and 3q [82]. The subtelomeres function in the process of chromosome recognition and pairing during meiosis and are also involved in maintaining chromosomal stability and regulation of gene expression [83,84,85]. The subtelomeric homologous sequences prevent heterochromatin spreading into neighboring gene-rich regions to prevent suppression of the genes within those segments [84].

## 3. Telomere End Replication Problem

**Figure 4:**
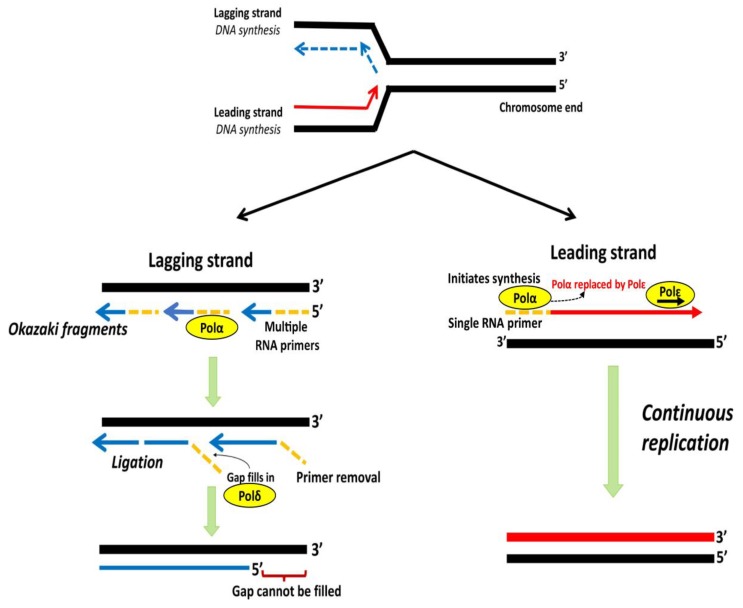
Schematic representation of lagging and leading strand replication. DNA polymerase Polα with a single RNA primer initiates synthesis of leading strand, which is subsequently replaced by Polε for further elongation. The lagging strand is copied through discontinuous Okazaki fragments from multiple primers. RNA primers are degraded and the gaps filled by Polδ followed by ligation of discontinuous fragments. The gap at 5′ end remains unfilled, leading to a non-replicated terminal region. Adapted from [7].

Incomplete replication at chromosomal ends by DNA polymerase results in progressive shortening of telomeres with each successive cell division and is termed as the “end replication problem” [1]. During DNA replication, a semi-conservative process, each DNA strand of a double helix acts as a template for the generation of a new complementary strand [7]. DNA polymerase Polα with a single RNA primer initiates the synthesis of a new strand in 5′ to 3′ direction towards replication fork, which is subsequently replaced by Polε for further elongation, forming the “leading strand” [86,87]. The synthesis of the “lagging strand” in the 5′ to 3′ direction requires annealing of multiple primers that elongate into short Okazaki fragments opposite to the replication fork and occurs less efficiently than the leading strand [88,89]. On completion of replication, the primer degradation results in internal gaps, filled by the polymerase, Polδ, and ligated to form a continuous strand. The gap left by the primer degradation at the terminal end remains unfilled, which results in the loss of a short segment of DNA at the 5′ end of the lagging strand [89]. The lagging strand synthesis fails to replicate an average length of ~250 nucleotides at the end of linear templates, which is hypothesized due to an inability of DNA Polα-primase to initiate lagging strand synthesis from the very end of linear DNA [90]. The loss of nucleotides at the chromosomal end leads to the G-rich single strand (Figure 4) at the end of the telomeres and, according to one hypothesis, the size of the 3′overhang is determinant of the rate of telomere shortening [91].

Normal human cells in a culture stop dividing after 40 to 60 passages, a phenomenon first observed by Leonard Hayflick and eponymously called the Hayflick limit [89,92]. Incomplete replication with a gradual shortening of telomeres acts as a counting mechanism that eventually leads to the replicative senescence [93]. On average, a single human telomere contains enough repeats to buttress the effect of telomere erosion in the absence of a maintenance mechanism, with an estimated loss of about 50 to 250 bp per mitosis [12,90,94]. Telomere shortening, to an extent, in proliferating cells of self-renewal tissues, such as hematopoietic cells, cells of the skin, and cells from gastrointestinal epithelium, is mitigated by holoenzyme telomerase [5,12,95]. Most of the adult stem cells and somatic tissues do not contain sufficient telomerase to maintain telomere length infinitely and therefore undergo age-related telomere shortening [96].

## 4. Mechanisms of Telomere Maintenance

The ribonucleic protein, telomerase, counteracts the replication-related telomere attrition. Telomerase is upregulated in tumors from over 90% of cancers; in 10% to 15% of tumors, telomeres are elongated through a homologous recombination-based alternative lengthening of telomeres (ALT) [97].

### 4.1. Telomerase Structure and Biogenesis

**Figure 5:**
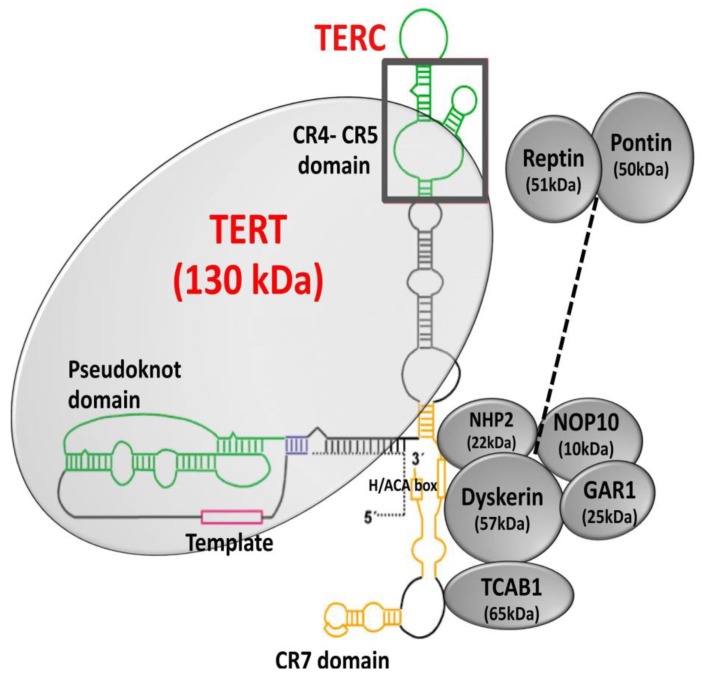
Structure of telomerase. Telomerase is a holoenzyme composed of the catalytic subunit, TERT (telomerase reverse transcriptase), and the RNA component, TERC (telomerase RNA component). Dyskerin and other associated proteins, GAR1, NHP2, and NOP10 interact with TERC by binding to the H/ACA box and regulate telomerase biogenesis, subcellular localization, and function. Adapted from [109,110].

Telomerase consists of a catalytic subunit, telomerase reverse transcriptase (TERT), and an RNA component (TERC), which acts as a template for the extension of telomeric nucleotide repeats [6,98,99]. A number of accessory molecules regulate telomerase biogenesis, subcellular localization, and function [100,101,102,103]. The 3′ end of TERC contains a conserved H/ACA domain (Figure 5) that binds the protein complex formed by dyskerin (DKC1), nucleolar protein 10 (NOP10), non-histone protein 2 (NHP2), and encoding H/ACA ribonucleoprotein complex subunit 1 (GAR1) [17,103,104]. NOP10 and GAR1 bind to dyskerin, and NHP2 binds to the RNA directly [105]. TERC in the nucleolus assembles with TERT to form a mature telomerase complex, followed by recognition of the Cajal body (CAB) box by telomerase and telomerase cajal body protein 1 (TCAB1), which in turn recruits mature telomerase complex to Cajal body [106]. During the S-phase of the cell cycle, Cajal bodies facilitate the recruitment of the mature telomerase complex to the telomeres [107]. Further, auxiliary proteins, such as ATPases reptin and pontin, have shown to be involved in telomerase assembly by interacting with TERT and dyskerin [108]. Pontin and reptin facilitate the assembly of TERT with TERC and dyskerin or remodel the mature telomerase complex. Through their interaction with dyskerin, pontin and reptin are involved in assembling and stabilizing TERC [108].

Telomerase activity remains tightly controlled at multiple levels- from transcriptional regulation of components for biogenesis to recruitment to the telomeres [111,112]. The model of repeat-addition processivity involves the addition of telomere repeats by the holoenzyme in successive steps without primer dissociation and requires several elements [12]. The number of repeats added by telomerase remains a controlled phenomenon with a set equilibrium and any disruption becomes causal for different telomere related diseases [12]. A number of proposed models have explained telomere length homeostasis [113]. The protein counting model predicated on telomere-bound proteins acting to block telomerase from a distance, with large numbers exerting a larger repressive effect and preferential elongation of shorter telomeres [114,115]. Another probabilistic model suggested the telomere length homeostasis via a switch between telomerase-extendible and telomerase non-extendible states, with a preferential shift towards the former state in short telomeres [116,117]. The replication fork model accounts for both negative regulation and preferential elongation of short telomeres with bound proteins exerting a negative effect that there would be increase in the probability of telomerase dissociation from the replication fork on short telomeres to reach the end for catalytic elongation [113].

### 4.2. Telomerase Reactivation

Telomerase reactivation occurs in tumors via multiple genetic and epigenetic mechanisms that include *TERT* and *TERC* amplification, genomic rearrangement of *TERT*, somatic mutations within the *TERT* promoter, and epigenetic modifications through *TERT* promoter methylation [97,118].

#### 4.2.1. Gene Amplification of TERT and TERC and Rearrangement of TERT

**Figure 6:**
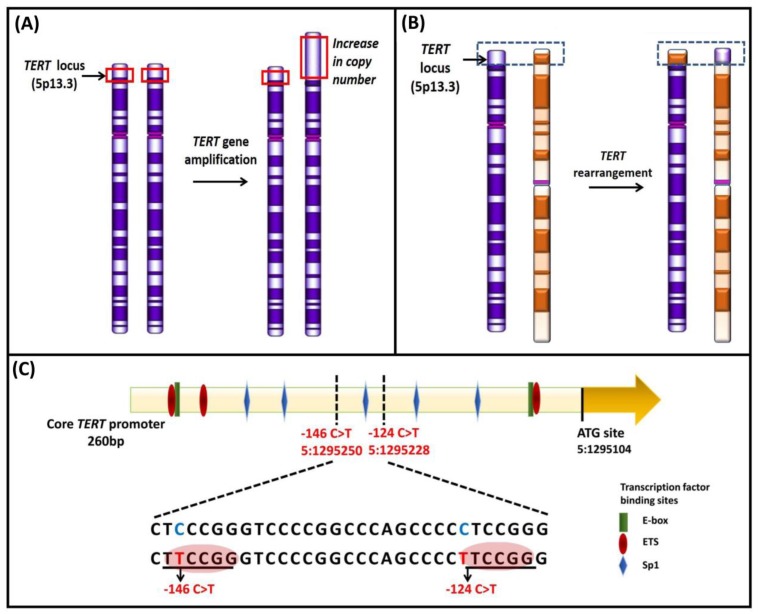
Schematic representation of genetic mechanisms of telomerase activation. (**A**) *TERT* gene amplification leading to an increase in *TERT* copy number at the 5p15.33 locus; (**B**) genomic rearrangement in *TERT* result in inter-chromosomal translocation; (**C**) Mutations at two hotspots in the *TERT* promoter, −124 and −146 bp from ATG start site create de novo binding sites for ETS transcription factors (red circles). Various transcriptional binding elements are represented in the core promoter region, E-box (green), ETS (red), and Sp1 (blue). Adapted from [12,97,145].

The regions containing the *TERT* gene at chromosome 5p15.33 and the *TERC* gene at chromosome 3q26.3 (Figure 6A) are frequently amplified in cancers [119]. *TERT* expression based on correlation with the gene dosage has been shown to be haploinsufficient for telomere maintenance [119,120,121,122]. In a systematic analysis of *TERT* gene amplification based on 31 tumor types from 6835 patients, *TERT* amplifications were observed in 4% of tumors [118]. *TERT* amplifications were frequent in ovarian cancer, adrenocorticol carcinoma, esophageal cancer, lung adenocarcinoma, and squamous cell carcinoma. Overall, only in 3% of tumors, increased *TERT* expression was attributed to the amplifications; other tumors involved diverse mechanisms [118]. Increased *TERT* gene copy number was associated with upregulation of the gene expression and correlated with worse clinical outcomes in breast, lung adenocarcinoma, Merkel cell carcinoma, and thyroid carcinoma [123,124,125,126]. In systematic analyses, *TERC* amplifications leading to an increased expression occur in about 4% of the tumors, which included lung squamous cell carcinoma, esophageal cancer, and ovarian cancer [118].

Another mechanism of *TERT* upregulation, observed in neuroblastoma, comprises genomic rearrangements (Figure 6B) affecting the *TERT* locus at 5p15.33 [127,128]. The rearrangements mainly cluster in a region 50 kb upstream of the *TERT* transcriptional site, leading to the juxtaposition of active super-enhancers in close proximity to the *TERT* locus that causes chromatin remodeling and consequent increased expression [127,128]. The *TERT* rearrangements occur mainly in high-risk neuroblastoma in mutually exclusiveness with *MYCN* amplifications and *ATRX* mutations [127,128].

#### 4.2.2. TERT Promoter Mutations

*TERT* promoter mutations represent frequent somatic genetic alterations that drive *TERT* expression and telomerase reactivation [12,129]. The recurrent somatic mutations within the *TERT* promoter mainly at −124 and −146 bp from the ATG start site generate de novo binding sites for E-twenty-six/ternary complex (ETS/TCF) transcription factors [130,131]. Other somatic *TERT* promoter mutations that create identical binding sites for ETS/TCF transcription factors include that detected at −57 bp, originally discovered as the causal germline mutation in a melanoma pedigree, and at −124/−125 bp and −138/−139 bp as CC > TT tandem mutations that occur mainly in skin cancers [130,132,133,134]. In glioblastoma, liver cancer and bladder cancer cell lines, GA binding protein transcription factor subunit alpha (GABPA) as in a heteromeric complex with GABPB1, binds to the *de novo* E-twenty-six (ETS) binding sites created by the *TERT* promoter mutations (Figure 6C) in cooperation with in-proximity native sites [135]. *TERT* promoter mutations occur mainly in cancers arising from tissues with low-rates of self-renewal that include glioblastoma, melanoma, urothelial carcinoma, squamous cell carcinoma, medulloblastomas, and aggressive thyroid carcinoma subtypes [12,131,136,137,138,139,140,141,142,143]. *TERT* promoter mutations contribute to tumorigenesis in a two-step mechanism. Those mutations during the initial phase, instead of preventing bulk telomere shortening, extend the cellular lifespan by stabilizing the shortest telomeres. In the second phase, the critically short telomeres lead to genomic instability and telomerase is further upregulated to sustain cell proliferation [144].

#### 4.2.3. Epigenetic Mechanisms

**Figure 7:**
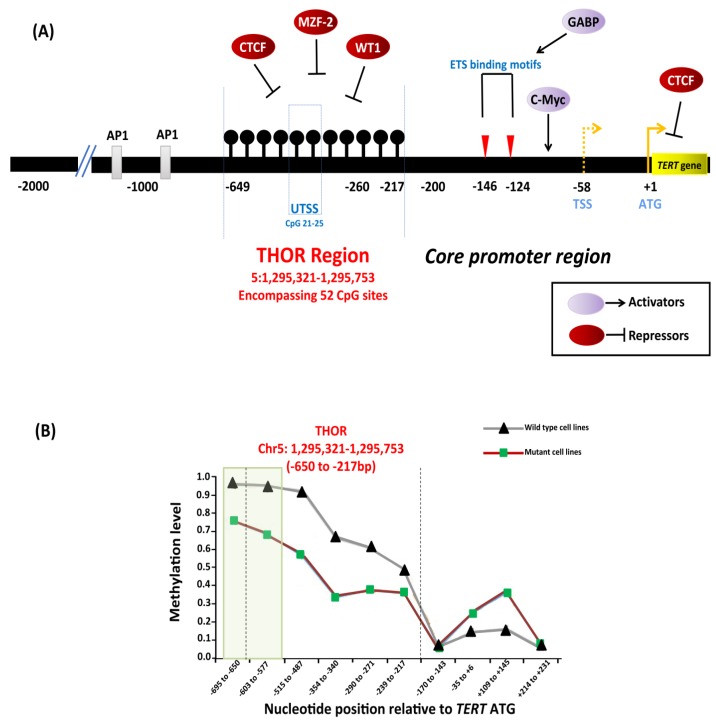
Epigenetic regulation of *TERT* in cancers. (**A**) Depiction of transcription factors along with binding sites, *TERT* promoter mutations −124C > T and −146C > T, hypermethylated region upstream of transcription start site (THOR). Binding of transcriptional activators, c-Myc, and repressors, CCCTC-binding factor (CTCF), myeloid zinc finger protein-2 (MZF-2), and Wilms tumor 1 (WT1) to the *TERT* promoter is controlled by DNA methylation as methylated CpGs prevent the binding to the target sites leading to *TERT* activation. The black lollipops represent methylated CpG sites. (**B**) Relative DNA methylation in tumor-derived cell lines with and without *TERT* promoter mutations. The green box represents a specific region in THOR (−668 to −577 bp relative to ATG) that is shown to be less methylated in cell lines with *TERT* promoter mutations than in cell lines without mutations. Adapted from [123,146,147].

The *TERT* gene contains a CpG island that extends from −838 bp from the ATG start site to a position near the end of exon 2 (Chr 5: 1,296,000–1,293,450) [146]. Conventional and next generation sequencing studies characterized a 433 bp genomic region within the promoter, extending from −650 to −217 bp from the ATG start site (GRCh37/hg19, Chr5: 1,295,321–1,295,753), that encompasses 52 CpG sites known as the *TERT* hypermethylated oncological region (THOR) [147]. THOR is hypermethylated in malignant tumors and hypomethylated in normal tissues and stem cells. DNA methylation controls the binding of transcriptional activators, c-Myc, and repressors CCCTC-binding factor (CTCF), myeloid zinc finger protein-2 (MZF-2), and Wilms tumor 1 (WT1) to the *TERT* promoter (Figure 7A). Hypermethylation prevents binding of the repressors to the promoter that leads to *TERT* upregulation and telomerase activation [123,147,148]. THOR methylation has been reported to have a diagnostic and prognostic role in pediatric brain tumors and prostate cancer [149,150].

Reduced methylation in the *TERT* promoter occurs in cancers that harbor *TERT* promoter mutations [146,151,152]. A specific region within the THOR, from −668 to −577 bp from the ATG start site (Chr5: 1,295,681–1,295,772), was shown to be hypomethylated in tumor-derived cell lines (Figure 7B) with *TERT* promoter mutations compared to those without mutations [146]. In the cell lines with *TERT* promoter mutations, the methylation was shown to be allele-specific, and H3K27me3 and H3K9me3 histone marks of inactivation promote the methylation [146]. The binding of the GABPA/B1 complex to the de novo sites on the mutant alleles causes an epigenetic change from an inactive H3K27me3 to an active chromatin mark H3K4me2/3, resulting in monoallelic expression [146,153]. The enzyme enhancer of zeste homolog 2 (EZH2), catalytic subunit of polycomb repressive complex 2 (PRC2), is responsible for the deposition of H3K27me3. The causal relationship between DNA and histone methylation was further supported by a strong binding preference for PRC2 at the methylated *TERT* promoter in vitro [146].

### 4.3. Alternative Lengthening of Telomeres

**Figure 8:**
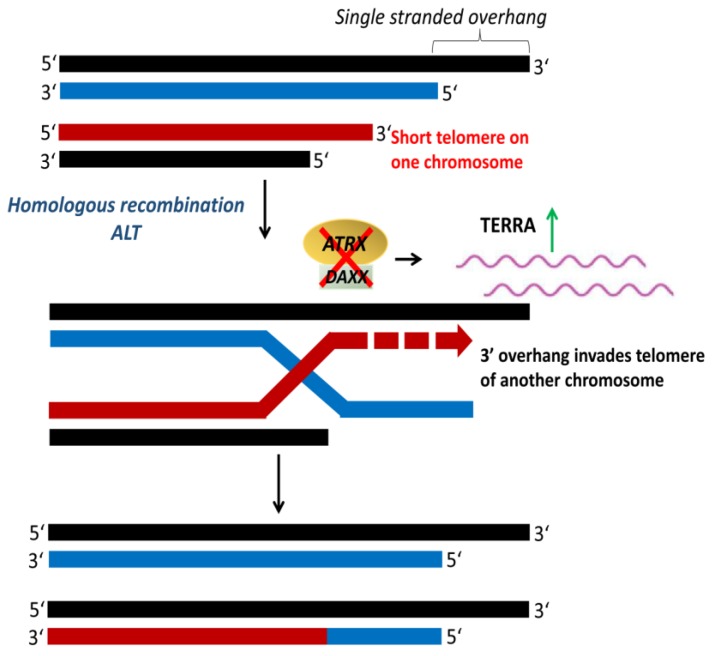
Alternative lengthening of telomeres (ALT). ALT is a telomerase-independent mechanism that occurs via homologous recombination to maintain telomere length. The inactivation of α-thalassemia/mental retardation syndrome X-linked protein (ATRX) and death domain-associated protein (DAXX) upregulates telomeric repeat containing RNA (TERRA), which activates telomeric recombination and initiation of ALT. Adapted from [97].

Cancer cells that maintain their telomeres by ALT (Figure 8) are characterized by heterogeneous telomere length with extremely long (>50 kb) and short (<5 kb) telomeres [154,155]. Telomeres in ALT cells cluster around promyelocytic leukemia (PML) nuclear bodies, referred to as ALT-associated PML bodies (APB) [156]. ALT is usually detected by telomere-specific fluorescence in situ hybridization, APB immunofluorescence, and ALT-associated molecule detection assays [157,158]. Mutations in the genes encoding for the α-thalassemia/mental retardation syndrome X-linked protein (*ATRX*) and the death domain-associated protein (*DAXX*) have been associated with ALT-positive tumors [159]. *ATRX*, together with *DAXX*, function as a chromatin remodeling complex that facilitates the deposition of histone variant H3.3 at the telomeres [160]. The loss of *ATRX* and *DAXX* due to mutations leads to a repressed heterochromatin state that activates recombination and initiation of ALT [154,161]. *ATRX* loss compromises the cell cycle regulation of TERRA and leads to the persistent association of replication protein A (RPA) with telomeres, resulting in a recombinant nucleoprotein structure [162]. ALT is observed at a high frequency in tumors of the central nervous system, peripheral nervous system, and sarcoma, but rare in carcinomas [159,163].

## 5. Telomere Length Heritability

Epidemiological studies have shown telomere length as a complex heritable trait with estimated heritability derived from twin studies from 36% to 82% compared to 34% to 50% from familial studies [164,165,166]. The predominant environmental factors shared between twin-pairs impact the telomere length during initial growth and development [164]. The two potential sources of heritability are inherited genetic variations that influence telomere maintenance and variability in telomere length per se [165,167,168].

The variability in telomere length in parental gametes is directly expressed in the offspring zygotes, but a correlation between offspring and paternal telomere length or offspring and maternal telomere length is not clear [165,169]. In a meta-analysis involving six different populations with 19,713 subjects, a high heritability estimate of 70% and a statistically significant correlation between maternal and offspring telomere was reported, which was attributed to an X-linked mechanism and mitochondrial DNA [170,171]. The effect of paternal age at conception on offspring telomere length has been widely reported in several studies, with evidence suggesting that newborns with older fathers had statistically significant long telomeres [170,171,172,173,174].

Telomere length inter-individual variation arises early in life due to an interplay between genetic and environmental factors [175,176]. Several genetic variants associated with telomere length have been identified through genome-wide association studies (GWAS), which to some extent account for inter-individual variation in telomere length in the general population [177,178,179,180,181,182,183]. In addition, the impact of environmental factors influencing telomere length during growth and development is also relevant to telomere heritability estimates [169].

### 5.1. Genetic Factors Associated with Telomere Length

A number of telomere length associated genetic loci associated have been identified through linkage analysis and GWAS [178,179,180,181,182,183,184,185,186]. In a study conducted on 383 adult subjects from 173 families, comprising of 258 sibling pairs, the first locus associated with mean telomere length was mapped to chromosome 12p11.2 [187]. In another linkage study with 1025 dizygotic twin pairs, chromosome 14q23.2 and two additional suggestive loci at 10q26.13 and 3p26.1 associated with telomere length were identified [188]. In a linkage analysis carried out on 4289 individuals from 586 families, with evidence for longevity, two additional loci were mapped at 17q23.2 and 10q11.21 [184].

Twelve GWAS (Table 1) on telomere length conducted on different study populations have been reported so far [178,179,180,181,182,183,184,186,189,190,191,192]. In a first GWAS conducted on 1625 women from the UK adult twin registry, two single-nucleotide polymorphisms (SNPs) represented by rs2162440 and rs7235755 on chromosome 18q12.2 were shown to be associated with telomere length, which could not be replicated in additional 1165 men and women from the UK twin registry [189]. First, statistically significant associated SNPs with telomere length were at 3q26, represented by rs12696304 and rs16847897, identified through GWAS on 1487 individuals with coronary heart disease and 1430 healthy controls with association replicated in independent cohorts [179,186]. In a meta-analysis on 3417 individuals from four populations, telomere length associated SNPs were identified within the segments with genes *OBFC1* and chemokine C-X-C motif receptor 4 (*CXCR4*) [181]. Following the initial discovery of SNPs in the *TERC* and *OBFC1* loci, several GWAS have identified additional variants associated with telomere length in loci containing *TERT*, *CTC1*, *NAF1*, and *RTEL1* [178,179,180,181,182,183,186].

Through a large-scale GWAS based on 26,089 healthy controls from breast, ovarian, and prostate cancer, four loci with telomere length associated SNPs were identified, including 3q26.2 (*TERC*), 5p15.33 (*TERT*) and 10q24.3 (*OBFC1*), and at chromosome 3p14.1 with the *PXK* gene [178]. So far, through GWAS, nine different loci telomere length associated variants have been identified. The individual SNPs in those genes exert only a small effect on telomere length; the combined effect of numerous such polymorphisms can be substantial [193].

**Table 1:** Telomere length-associated single nucleotide polymorphisms.

Genes (Locus)	SNP ^a^	Genomic Position (GR37/hg19) ^b^	ΔTL ^c^	*p*-Value ^d^	Reference
*TERC* (3q26.2)	rs1317082 rs10936601 rs12696304 rs16847897 rs10936599 rs3772190	3:169497585 3:169528449 3:169481271 3:169568116 3:169492101 3:169500487	(−) 77 bp NA (−) 75 bp (−) 33 bp NA NA	1 × 10^−8^ 4 × 10^−15^ 4 × 10^−14^ 1 × 10^−5^ 3 × 10^−31^ 2 × 10^−1^	[178] [178] [179] [179] [180] [186]
*OBFC1* (10q24)	rs2487999 rs9420907 rs4387287 rs9419958	10:105659826 10:105676465 10:105677897 10:105675946	(+) 100 bp NA (−) 230 bp NA	4 × 10^−14^ 7 × 10^−11^ 2 × 10^−11^ 9 × 10^−11^	[178] [180] [181] [182]
*TERT* (5p15.3)	rs7726159 rs2736100 rs2736108 rs7705526 rs2853669	5:1282319 5:1286516 5:1297488 5:1285974 5:1295349	(+) 73 bp (−) 94 bp NA (+) 90 bp (+) 60 bp	5 × 10^−17^ 4 × 10^−6^ 5 × 10^−5^ 1 × 10^−15^ -	[178] [180] [194] [194]
*PXK* (3p14.4)	rs6772228	3:58376019	(−) 120 bp	4 × 10^−10^	[178]
*ZNF311* (6p22.1)	rs9257445	6:28949206	(−) 38 bp	1 × 10^−7^	[178]
*BCL2L1* (20q11.2)	rs6060627	20:30262159	(+) 36 bp	6 × 10^−7^	[178]
*GRIA4* (11q22.3)	rs610160	11:105696895	(+) 30 bp	7 × 10^−6^	[179]
*NAF1* (4q32.2)	rs7675998	4:164007820	(−) 90bp	4 × 10^−16^	[180]
*RTEL1* (20q13.3)	rs755017	20:62421622	(−) 74 bp	7 × 10^−9^	[180]
*ACYP2* (2p16.2)	rs11125529	2:54475866	(−) 67 bp	8 × 10^−10^	[180]
*ZNF208* (19p12)	rs8105767	19:22215441	(−) 58 bp	1 × 10^−9^	[180]
*MPHOSPH6* (16q23.3)	rs2967374	16:82209861	NA	3 × 10^−7^	[180]
*CTC1* (17p13.1)	rs3027234	17:8136092	(−) 57 bp	2 × 10^−8^	[182]
*ZNF676* (19p12)	rs412658	19:22359440	(−) 49 bp	1 × 10^−8^	[182]
*DCAF4* (14q24.2)	rs2535913	14: 73415233	(−) 45 bp	2 × 10^−7^	[195]
*DHX35* (20q11.23)	rs6028466	20:38129002	NA	3 × 10^−7^	[183]
*DKK2* (4q25)	rs7680468	4:108304199	NA	5 × 10^−8^	[184]
*CSNKA2* (16q21)	rs74019828	16:58209274	(−) 38 bp	5 × 10^−8^	[190]

^a^ SNP, single nucleotide polymorphism. ^b^ Genomic position of SNP from GRCh37/hg19 reference genome. ^c^ Differences in telomere length estimates (given in base pairs, bp) for the variant allele of each SNP associated with telomere length, determined from GWAS. NA, data not available. ^d^ *p*-values from GWAS summary data showing genome-wide statistical significance.

#### Functionality of Telomere Length-Associated Single Nucleotide Polymorphisms

The functional impacts of the SNPs rs3027234 and rs2535913 at the loci 17p13.1 and 14q24.2 associated with telomere length were assessed from genome-wide expression data [182,195]. The minor allele (T-allele) of the SNP rs3027234, located in intron 11 (GRCh37/hg19 Chr17: 8,136,092) of the *CTC1* gene, associated with low expression of the gene [182]. CTC1 is a component of the telomere-binding CST complex, which binds to the telomeric 3′ single strand and functions to promote replication by stimulating Polα-primase activity [180,196]. Reduced expression of *CTC1* impairs complex formation with *STN1* and *TEN1* [197,198]. Depletion of the CST complex results in insufficient accumulation of Polα for efficient replication at the telomeres, leading to progressive telomere attrition [198].

The SNP rs2535913, located in intron 8 (GRCh37/hg19 Chr14: 73,415,233) of the DDB1 and CUL4-associated factor 4 (*DCAF4*) gene, leads to the reduced gene expression by affecting the binding of CTCF and Rad21. *DCAF4* forms a complex with *DDB1* and *CUL4* that is involved in nucleotide excision repair [195]. Rad21, a component of the cohesion complex, and CTCF have been implicated in telomere maintenance [195,199]. The depletion of CTCF or Rad21 results in reduced binding of TRF1 and TRF2 to telomeres [200]. *DCAF4* indirectly influences telomerase activity and telomere length through its interaction with DDB1. *DDB1* functions as a binding partner for the transcription factor E2F1, a member of the E2F family of transcription factors that regulate cell proliferation and telomerase activity [201,202]. The exact function of *DCAF4* on telomere regulation remains unclear [203]. In another functional study, the minor allele of the SNP rs2630578 located in intron 1 (GRCh37/hg 19 Chr12: 32,305,787) was shown to be associated with a reduced mRNA expression level of Bicaudal D Homolog 1 (*BICD1*), which functions in vacuolar traffic and regulates telomere length via telomerase and Ku-protein pathways [185,204]. The region surrounding the SNP exhibited the heterochromatin mark, H3K4me3, and the minor allele was shown to disrupt a putative binding sequence for Nuclear Factor Y (NF-Y) transcription factor, which is essential for *TERC* expression [185,205].

In addition, SNPs at chromosome 5p15.33 and 3q26.2, not associated with telomere length, were shown to affect *TERT* and *TERC* expression, respectively. The genomic region on chromosome 5 at 5p15.33, harboring *TERT* and cleft lip and palate associated transmembrane 1-like protein (*CLPTM1L*) genes, has been reported to contain several independent cancer susceptibility loci [194,206,207]. Fine-mapping analysis of the region in GWAS from four cancers identified an SNP, rs36115365, with a functional role in regulating *TERT* expression, which is located in-between the 5′end of *TERT* and 3′ end of *CLPTM1L*, with active histone modification marks and multiple transcription factor binding sites [208]. Transcriptional silencing of the regulatory region, encompassing the SNP, results in reduced telomerase activity and telomere length. The transcriptional regulator Zinc finger transcription factor 148 (*ZNF148*) preferentially binds to the minor allele of the variant that mediates increased *TERT* expression [208]. In a study based on 3912 individuals from the general population, the rs2293607 variant at 3q26.2, harboring the *TERC* gene was shown to alter the secondary structure of *TERC* mRNA, with the minor allele associating with an increase in the gene expression and telomere length [209].

The functional studies provide a framework for a genetic approach to investigate the causal role of telomere length in age-associated diseases [182,185,195]. However, to establish a causal link between the genetic variants associated with telomere length and disease risk is particularly challenging because other environmental and lifestyle factors also affect telomere length [169].

### 5.2. Environmental Factors Affecting Telomere Length

Several other factors that influence telomere length include oxidative stress, inflammation, lifestyle factors, physiological stress, and exposure to carcinogens [210,211,212]. The association between telomere length and environmental, occupational, and health risk factors has been reported in several cross-sectional epidemiological studies [210,211,212]. Oxidative stress is reportedly one of the most important causes of telomere shortening and reflects an imbalance between antioxidants and reactive oxygen species (ROS) [213,214]. Telomeres, due to high guanine content, are targets of oxidative damage through the formation of 8-hydroxy-2-deoxyguanosine (8-oxodG), an important marker of oxidative stress, which causes accelerated shortening [214,215]. Single-stranded breaks preferentially accumulate at telomeres in conditions of mild oxidative stress, which cause replication fork stalling and incomplete replication of chromosome ends, again leading to telomere shortening [216]. Environmental exposure to ultraviolet and ionizing radiation and exposure to carcinogens such as arsenic and lead cause DNA damage either directly or indirectly through the induction of oxidative damage or onset of chronic inflammation [192,215,217,218,219,220].

Other lifestyle factors like smoking, obesity, and lack of exercise increase the rate of telomere shortening. In a meta-analysis based on 84 studies, it was reported that smokers had shorter telomeres than non-smokers [221]. Various aspects of socio-economic status, particularly educational attainment and social support, have shown to influence telomere length [222,223]. In a study based on 84,996 non-Hispanic whites, individuals with low socio-economic status had short telomeres [224].

## 6. Telomere Length and Risk of Cancers

The association between telomere length and risk of cancers has been reported in several epidemiological studies [225,226,227,228,229]. Studies conducted in large cohorts have consistently demonstrated an association between increased telomere length and risk of various cancers, including melanoma, basal cell carcinoma, glioma, lung cancers, tumors of the urogenital system, and lymphoma [226,230,231,232]. The genetic basis for those observations is provided through Mendelian randomization and studies showing that various polymorphisms that modulate telomere length also affect the risk of different cancers, with alleles segregating with long telomeres associating with increased risk [226,230,231]. Within the cellular context, long telomeres afford increased proliferative potential until the replicative crisis and the telomere length acts as a deterministic factor in cancer development [144,233]. Different investigations over the years, in contrast, have suggested that short telomeres associate with poor patient survival in different cancers [234,235,236,237,238]. Extremely short telomeres, caused by defective components that either protect or elongate telomeres due to genetic mutations, result in various debilitating disorders, referred to as telomeropathies [239,240,241].

In a Mendelian randomization study on 22 primary cancers involving 4,20,081 cases and 10,93,105 controls, genetically increased telomere length was shown to be associated with increased risk of nine cancers, which included glioma, serous low-malignant potential ovarian cancer, lung adenocarcinoma, neuroblastoma, bladder cancer, melanoma, testicular germ-cell cancer, kidney and endometrial cancer [226]. Those findings were similar in direction and magnitude of risk estimates reported previously in observational and Mendelian randomization studies [142,193,225,227,228,229,230,231,242,243,244,245,246,247]. Although long telomeres have been consistently reported to show statistically significant association with increased risk of various cancers, some conspicuous exceptions to that generalization have been reported in different studies [226,230,231,248,249]. In a Mendelian randomization study based on 2374 pancreatic cancer cases and 4326 controls, genetically decreased telomeres were associated with increased risk of pancreatic cancer [248]. Exposure to carcinogens such as arsenic has been shown to modulate the direction of the effect of telomere length on cancer risk. In a study on basal cell carcinoma, a reversal of the effect was reported, where arsenic exposure and short telomeres were shown to synergistically increase the risk in a dose-dependent manner [249].

## 7. Telomeres as Potential Targets for Anti-Cancer Therapy

Telomeres and telomerase-based therapies are emerging as prospective cancer treatment strategies [250]. Telomerase inhibitors such as small molecule inhibitors, antisense nucleotides, G-quadruplex stabilizers, *TERT*-dependent anticancer immunotherapy, and chemical inhibition of telomerase are the most commonly studied anti-cancer treatment strategies [251,252]. Telomeres are also targeted using guanine-rich oligonucleotide (GRO) homologous to the 3′ single-stranded overhang, known as T-oligos, a specific 11-base oligonucleotide sequence, (5′ GTTAGGGTTAG), which accumulates in the nucleus and induces DDR with minimal or no functional effect on normal cells [250,253]. Treatment with T-oligos in vitro has been shown to be effective in reducing viability and tumor growth in several cancers, including melanoma, prostate, ovarian, lung, breast, and colorectal cancer [250,254,255,256,257,258]. T-oligos are hypothesized to interfere with normal telomeric structure and form G-quadruplexes, inducing genomic stress in addition to aberrant upregulation of DDR pathways, and TRF2 and POT1 have shown to be upregulated after T-oligo treatment [250,254,259]. T-oligos induce DDR mechanism via two potential modes, the shelterin dissociation model (SDM) and the exposed telomere mimicry model (ETM). The SDM model proposes that the T-oligos upon introduction into the nucleus compromise the integrity of telomeres through the displacement of shelterin proteins, leading to the unfolding of t-loops and induction of DDR response. The ETM model proposes that T-oligos accumulate in the nucleus and are recognized as damaged telomeres, initiating a DDR mechanism similar to those occurring during excessive telomere shortening [250].

Telomere dysfunction mediated through telomerase substrate precursor, 6-thio-2′-deoxyguanosine (6-thio-dG), impairs cell viability and tumor growth [260]. 6-thio-dGTP, which is formed in cells from 6-thio-dG, gets recognized by telomerase and incorporated into telomeres leading to telomere dysfunction-induced foci in telomerase positive lung and colon cancer and *BRAF*-mutant melanoma cells [260,261]. Therapeutic inhibition of TRF1 binding to the telomeres using small molecules have been shown to suppress the growth of lung carcinomas and glioblastoma by inducing the DDR mechanism [262,263,264]. Imetelstat (GRN163L) directly targets telomerase by antagonistically binding to TERC; however, the long term effects are not known [265]. G-quadruplexes inhibit telomerase activity by blocking the binding of TERC [250]. The use of G-quadruplex stabilizers as treatment for progressive and malignant cancers gradually shortens the 3′ single-stranded ends of the telomeres, without reducing the overall length of the telomeres, thereby indirectly inhibiting telomerase activity [250,266]. Although the *TERT*-based therapeutic vaccination have limited anti-proliferation efficiency, the focus has shifted to personalized interventions specifically for patients with *TERT* promoter mutations and *TERT* genomic rearrangements, in combination with immune-checkpoint inhibitors [267]. Individuals with short telomeres are more prone to damage by irradiation compared to those with long telomeres. Telomerase inhibitors such as Imetelstat, coupled with radiotherapy, enhanced the cancer cell response to irradiation via telomere dysfunction [268]. Inhibitors directly targeting telomeres, such as T-oligos, G-quadruplexes, telomestatin, G-quadruplex ligand, and shelterin proteins-TRF2, TPP1, and POT1, are also shown to improve radiosensitivity [268,269].

## 8. Conclusions

Telomeres, the dynamic structures at chromosomal ends, are crucial for genomic integrity, and through age-dependent attrition, act as tumor suppressors [12]. Telomeres are protected from being recognized by DNA damage response by components of shelterin complex that also assists in the recruitment of telomerase for elongation of repeats [16]. Telomerase, a tightly regulated holoenzyme, while limited in most somatic cells, is upregulated as a major hallmark through different mechanisms in a majority of human cancers to impart unlimited replicative potential [5]. Telomere length per se, a hereditary trait, has been associated with different diseases, including various cancers. Extremely short telomeres, characteristics of various telomere diseases, are caused by genetic mutations in different components involved in telomerase function. Genetically-driven long telomeres, with some exceptions, in many studies, have been shown to increase the risk of different cancers. Telomeres not only represent functional segments in the human genome but also hold potential as targets for anti-cancer strategies.
